# Calcific Tendinitis of the Shoulder: A Neuro-Psychomotor Behavioral Diagnostic and Therapeutic Approach With Radioelectric Asymmetric Conveyer Neurobiological Stimulation Treatments

**DOI:** 10.7759/cureus.26770

**Published:** 2022-07-12

**Authors:** Vania Fontani, Arianna Rinaldi, Alessandro Castagna, Salvatore Rinaldi

**Affiliations:** 1 Research Department, Rinaldi Fontani Foundation, Florence, ITA; 2 Department of Regenerative Medicine, Rinaldi Fontani Institute, Florence, ITA; 3 Department of Adaptive Neuro Psycho Physio Pathology and Neuro Psycho Physical Optimization, Rinaldi Fontani Institute, Florence, ITA; 4 Biomedical Sciences, University of Sassari, Sassari, ITA

**Keywords:** neurobiological stimulation, biostimulation, regenerative medicine treatments, reparative medicine treatments, stress, anxiety, depression, piezoelectric effect, calcific tendonitis, calcific tendinitis of shoulder

## Abstract

Calcific tendinitis of the shoulder (CTS) is one of the pathological conditions that most often affects the shoulder and consists of a calcium deposit that settles within the tendon tissue of the rotator cuff. The scientific literature has long highlighted the impact of anxiety, stress, and depression on CTS. The goal of this case report is to highlight how the emotional state of patients and their neuro-psychomotor behavior induce a state of constant muscular tension which, through the physical phenomenon of piezoelectricity, causes calcium salts to precipitate and form calcifications. Therefore, stress, anxiety, and depression are likely factors underlying the etiopathogenesis of CTS. Consistent with this interpretation, this report presents five cases of CTS treated with three specific neurobiological stimulation treatments using the radioelectric asymmetric conveyer (REAC) technology, which has demonstrated its effectiveness on alterations in postural attitude intended as neuro-psychomotor behavior, anxiety, stress, and depression, as well as on autonomic and metabolic alterations of the tissues at a local level. The results presented suggest that this approach may be useful in the treatment and prevention of CTS.

## Introduction

Calcific tendinitis of the shoulder (CTS) is one of the pathological conditions that most often affects the shoulder [1]. Although it is a common condition, its underlying mechanism remains unknown [2]. CTS consists of a calcium deposit that settles within the tendon tissue of the rotator cuff. Epidemiologically, the CTS varies between 2.7% and 10.3% based on previous studies. In general, CTS is twice as common in women than in men. The radiological diagnosis of CTS usually follows symptomatic episodes of the shoulder, such as functional limitation, stiffness, and sudden pain, sometimes even intense during movements, especially localized to the rotator cuff. In about 5% of the population, CTS can evolve into the frozen shoulder (FS) or adhesive capsulitis [3]. Females are four times more often affected than men [3].

Various causes are hypothesized in the etiopathogenetic mechanism of the deterioration of the scapulohumeral joint, which represents the initial step of the CTS. The most common mechanisms invoked are aging, clothing lifestyle, sedentary life, and habitual movements but also factors such as hypertension, diabetes, alcoholism, smoking, and autoimmune pathologies for calcium dysmetabolism contribute to the weakening of the muscles and tendons because they reduce the blood supply [1]. To date, none of these causes have been confirmed with certainty as the real initial physiopathological mechanism of the CTS. Up to now, all the factors described above seem to be a corollary of the phenomenon of CTS rather than a triggering cause. This case report aimed to analyze how depression, anxiety, and stress [4,5] condition the neuro-psychomotor behavior of the subject and can be the initial cause of CTS. The analysis of this correlation suggests a behavioral neuro-psychomotor approach to the etiopathogenesis of the disease and its therapeutic treatment. In CTS, calcium deposits are noted in the tissues. Physics and the piezoelectric effect can provide an easy and justified key to understanding how this phenomenon of calcium in tissues can be determined [6]. Piezoelectricity is the property of some crystalline materials to polarize, generating an electric potential difference when subjected to mechanical deformation. Piezoelectricity is a characteristic of many tissues in our body. Due to this characteristic, known for many years [7], in biological tissues, such as collagen, subjected to variations in elastic tension, calcium salts precipitate favoring the formation of calcifications. This phenomenon is particularly noticeable at the level of bone and tendon tissues [8]. In living subjects, including human beings, the structures that contribute the most to determining load variation at the joint level are the muscles. With their contraction, they can sufficiently determine piezoelectric phenomena in the joint structures [9].

Naturally, muscles must maintain their contraction for a sufficiently long time to determine the organization of the precipitated calcium salts in a calcific formation. The mechanism that can most easily determine this situation in an unconscious way is the postural attitude, which is a neuro-psychomotor behavior correlated to the subject’s emotional state [5]. In the 70s, some studies [10] had highlighted correlations between CTS and emotional and behavioral states, such as depression and anxiety [5,11-13]. On these assumptions, our behavioral neuro-psychomotor etiopathogenic and therapeutic approach was developed. To deal therapeutically with CTS based on this approach, we used specific neurobiological stimulation treatments applied exclusively using the radioelectric asymmetric conveyer (REAC) technology. The first treatment used is called neuro-postural optimization (NPO). This treatment, which consists of a single administration, is specifically aimed at unblocking dysfunctional neuro-psychomotor adaptations, such as functional dysmetria, promoting a better functionality of the behavioral motor engrams [14,15]. The second treatment used is called neuro-psycho-physical optimization (NPPO). This treatment, which consists of a cycle of 18 sessions lasting a few seconds, has been designed to improve mood and behavioral disorders, such as depression, anxiety, and stress [13,16]. The third neurobiological stimulation treatment used is called tissue optimization type B (TO-B). This treatment also consists of a cycle of 18 sessions lasting a few minutes. The TO-B treatment has been specifically studied to correct those alterations in the tissues induced by an altered autonomic response, supported by emotional and behavioral states [17].

## Case presentation

Case one

**Figure 1:**
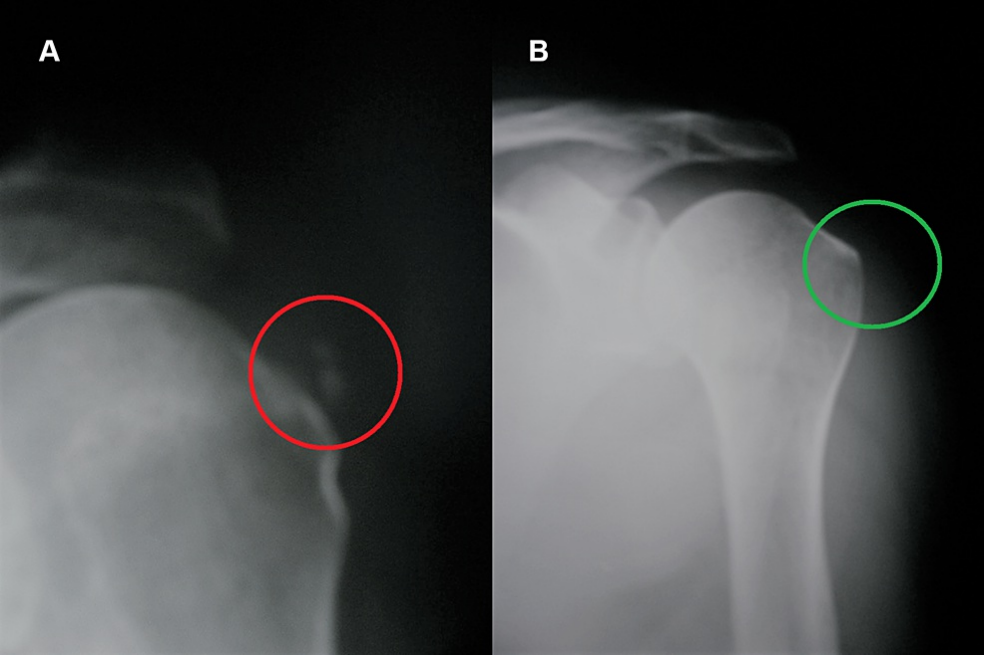
X-rays before and after REAC treatments. The calcifications are highlighted in red (A), and the disappearance of the calcification is highlighted in green (B). REAC: radioelectric asymmetric conveyer

The first case concerns a 60-year-old woman, a cook. During the first observation, in addition to complaining of local pain and functional impotence in the left shoulder, the patient complained of a state of strong anxiety and stress related to work overload. Clinical examination revealed shoulder calcific periarthritis of the left shoulder. This diagnosis was confirmed by the radiological examination performed before the REAC treatments, which showed minute multiple calcifications of the periarticular soft tissues (Figure 1, Panel A).

On a check-up three months after the end of the REAC treatments, the patient reported the disappearance of the local pain and restoration of the full functionality of the left shoulder. Moreover, the patient affirmed a clear improvement in the quality of life both in the workplace and in society, with a reduction in feelings of anxiety and stress. The radiological results showed the total disappearance of the calcifications after about three months from the previous X-rays (Figure 1, Panel B).

Case two

A 48-year-old construction worker presented to us because all previous treatments had been ineffective in relieving pain and improving the functionality of his right shoulder. The patient reported having suffered from depressive episodes treated pharmacologically, but as the drugs used negatively affected his skills at work, they were taken inconsistently and for short periods. The reported symptoms of vivid pain and functional impotence suggested a diagnosis of FS.

**Figure 2:**
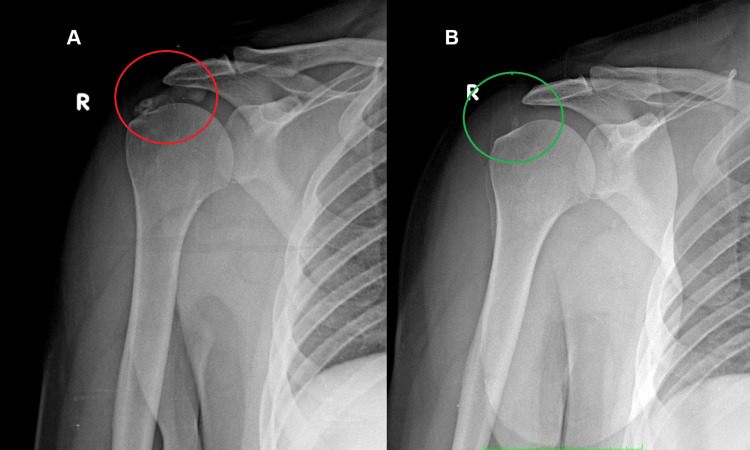
X-rays before and after REAC treatments. The calcifications are highlighted in red (A), and the disappearance of the calcification is highlighted in green (B). REAC: radioelectric asymmetric conveyer

Before the REAC treatments, the X-rays showed important multiple calcifications of the supraspinatus, confirming FS (Figure 2, Panel A).

On check-up four months after the REAC treatments, it is interesting to highlight that the patient, while reporting a full recovery of work activity with the disappearance of the pain in the right shoulder, affirmed as the main improvement the positive change in mood and the way he faced daily adversities. The X-rays showed almost total disappearance of the calcifications previously reported, with a total recovery of shoulder function (Figure 2, Panel B).

Case three

**Figure 3:**
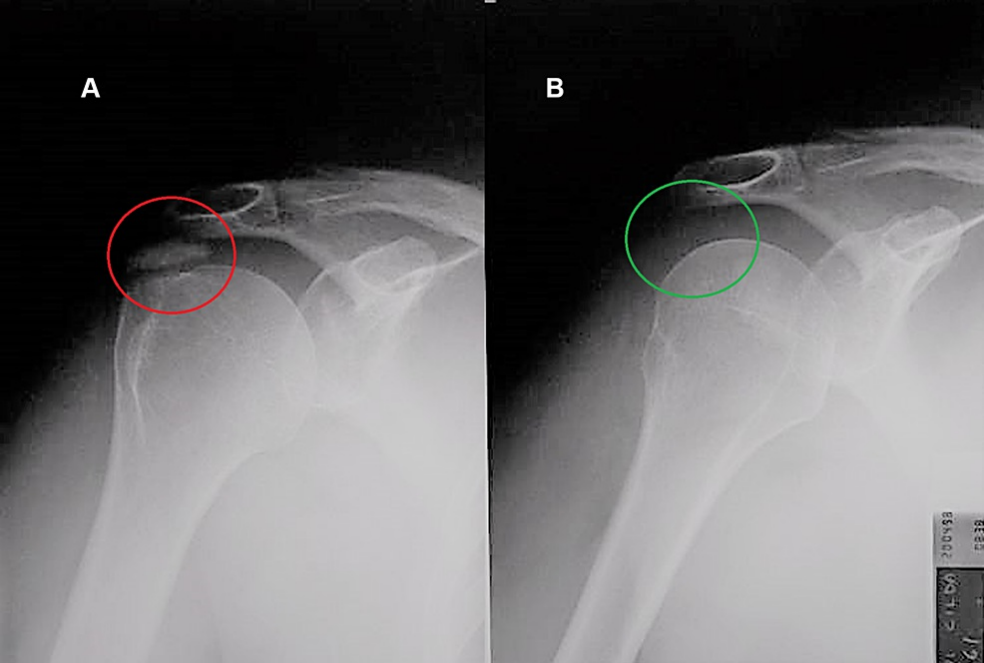
X-rays before and after REAC treatments. The calcifications are highlighted in red (A), and the disappearance of the calcification is highlighted in green (B). REAC: radioelectric asymmetric conveyer

The third case concerns a 53-year-old housewife. The patient reported that she had suffered for many years from pain and functional limitation in the right shoulder, which did not improve with any treatment, neither pharmacological nor rehabilitative. In addition, she reported widespread pain, so much so that she was diagnosed with suspected fibromyalgia. Previous X-rays showed a picture of major calcification of the cuff, particularly of the supraspinatus (Figure 3, Panel A).

At the follow-up visit three months after the end of the REAC treatments, the patient reported a reduction not only in shoulder pain but in all her pains generally, as well as an improvement in mood and night rest. The X-rays performed six months after the end of the therapy showed the total disappearance of the calcification (Figure 3, Panel B).

Case four

**Figure 4:**
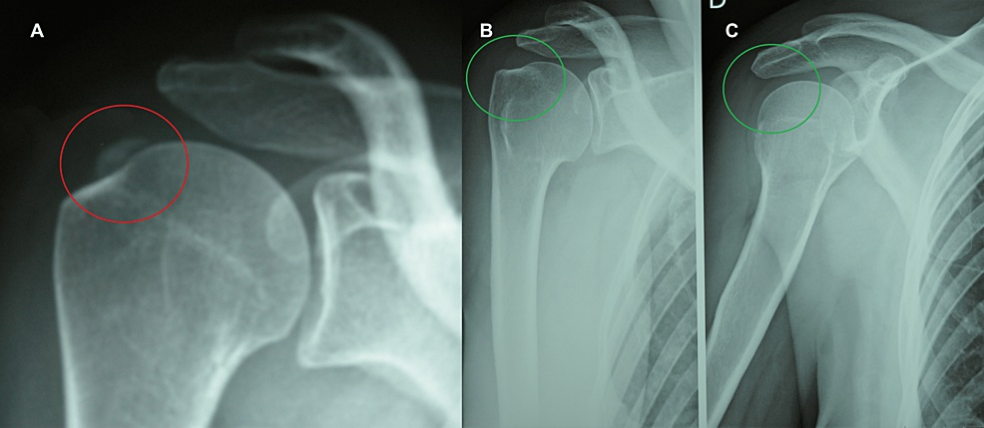
X-rays before and after REAC treatments. The calcifications are highlighted in red (A), and the disappearance of the calcification is highlighted in green (B-C). REAC: radioelectric asymmetric conveyer

The fourth case concerns a 70-year-old housewife. On first observation, she complained of local pain and functional impotence of the right shoulder. The patient reported that her symptoms worsened in relation to responses to anxiety and stress. The X-rays showed calcification of about 1 cm in size near the humeral trochitis (Figure 4, Panel A).

The patient was administered the REAC NPO, NPPO, and TO-B treatments. At the three-month follow-up, the patient reported resolution of the symptoms, with the disappearance of the pain and a resumption of the function of the right shoulder. Particularly, she affirmed that stress and anxiety episodes did not have a negative impact on her shoulder. About four months after the treatments, the X-rays showed the total disappearance of the previously reported calcification (Figure 4, Panels B, C).

Case five

**Figure 5:**
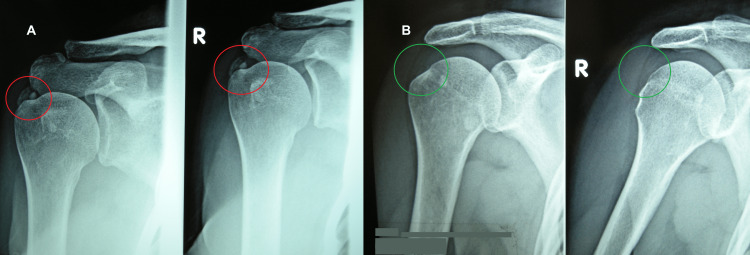
X-rays before and after REAC treatments. The calcifications are highlighted in red (A), and the disappearance of the calcification is highlighted in green (B). REAC: radioelectric asymmetric conveyer

The fifth case concerns a 68-year-old housewife. The patient, suffering for a long time from severe pain and functional impotence of the right shoulder, had undergone various unsuccessful treatments, such as infiltrative, analgesic, and rehabilitative treatments. According to the patient, this situation generated in her an anxious depressive state, not responsive to either psychotherapeutic treatment or antidepressant therapy. On first observation, she presented a clinical picture of FS with significant pain and functional impotence. The X-rays showed insertional calcifications of the rotator cuff and periarticular calcifications (Figure 5, Panel A).

On follow-up one month after the REAC treatments, the patient reported recovery of the joint function and pain disappearance. Thirty-five days after the end of the therapy, the X-rays showed a near disappearance of the calcifications (Figure 5, Panel B).

## Discussion

It might seem unlikely that a purely orthopedic pathology may have a psychogenic/neuropsychic origin or component that determines its onset or conditions and its severity. For some years, the scientific literature has been increasingly highlighting this correlation between psychogenic/neuropsychic components and orthopedic pathologies, such as calcific tendinitis of the shoulder, FS [13], or osteoarthritis of the knee [18]. Nevertheless, in clinical practice, this aspect is considered neither by the patient nor by the therapist who continues to use exclusively pharmacological and physiotherapy treatments.

The most common treatments for calcific tendonitis are non-steroidal anti-inflammatory drugs (NSAIDs), steroid injections, and physical therapy. In case of failure of the previous treatments, therapeutic ultrasound, extracorporeal shockwave therapy, radial shockwave therapy, joint washing treatment with saline solution, intra-articular infiltrations with hyaluronic acid, and surgical treatment are also used. The treatments listed above are intended to reduce pain, inflammatory processes, and mechanically or chemically break down calcification, but none of these are intended to reduce the constant state of muscle tension induced by depression, anxiety, and stress, conditions reported by various authors in the field of CST [4,5,10,13,16].

Based on the behavioral neuro-psychomotor approach, the constant muscular tension of the subject triggers a piezoelectric effect, which, in turn, determines and supports the formation and maintenance of calcification. Therefore, the first therapeutic goal is to act on the state of the behavioral neuro-psycho-physical tension of subjects, and consequently, on their state of constant muscle tension. Moreover, the altered behavioral state affects the autonomic response that conditions the microcirculation and metabolic processes at a local level. For these reasons, the remodeling of the autonomic components, capable of influencing the vascular, inflammatory, and immune response at the tissue level also represents further therapeutic support. The combination of REAC NPO, NPPO, and TO-B neurobiological stimulation treatments can represent a therapeutic arsenal useful in the prevention and treatment of CTS.

## Conclusions

The cases described in this study offer evidence of how useful the behavioral neuro-psychomotor approach can be as a key to understanding how CTS arises and which factors contribute to sustaining it. It would be opportune that the psychogenic/neuropsychic component be increasingly investigated in the context of orthopedic pathologies, analyzing how these components unconsciously condition the motor behavior patterns and determining osteoarticular pathologies over time.

REAC NPO, NPPO, and TO-B neurobiological modulation techniques allow the therapist to deal with the psychogenic/neuropsychic components of osteoarticular pathologies.

The REAC neurobiological modulation treatments used in this report allow for the optimization of the neuropsychic abilities and the autonomic response altered by psychogenic/neuropsychic states also at a local level, determining the reduction of the probably primary cause of joint damage.

The findings described in this manuscript highlight how the REAC neurobiological stimulation treatments can constitute an effective therapeutic strategy in preventing and treating CTS cases.
